# Effect of Supplementation with Antioxidants on the Quality of Bovine Milk and Meat Production

**DOI:** 10.1155/2013/616098

**Published:** 2013-11-21

**Authors:** Cristina Castillo, Víctor Pereira, Ángel Abuelo, Joaquín Hernández

**Affiliations:** Department of Animal Pathology, Veterinary Faculty, University of Santiago de Compostela, Campus Universitario, 27002 Lugo, Spain

## Abstract

From a clinical point of view, oxidative stress (OS) is considered the primary cause of numerous metabolic processes in *transition cow.* Thus, the addition of antioxidants has been considered a palliative or preventive treatment. But beyond the clinical perspective, antioxidant supplementation provides an added value to the product obtained being either milk or meat. This paper reviews the beneficial aspects that provide antioxidant supplementation on quality of both products and that fit into the new concept that the consumer has a functional and healthy food. Our approach is from a veterinary standpoint, by reviewing the studies conducted to date and the new perspectives that are interesting and need to be studied in the following years. One of the highlights is that sustainable farming, one in which production is combined with animal health, also impacts positively on the quality of the final products, with beneficial antioxidant properties to human health.

## 1. Introduction

Today, oxidative stress (OS) is considered a metabolic disturbance that affects organ systems and its presence will affect not only the health status of the animals but also the quality of the final products, such as milk or meat [1, 2]. However, under proper nutritional and environmental conditions, it increases the antioxidant defense, maintaining a balance that favors the pregnancy to term without compromising on maternal health. Recent research lines are aimed at strengthening the antioxidant defense in the transition phase to counteract the effects of many organic pathologies such as ketosis, hypocalcemia, and mastitis, among others [3]. The type of feed affects nutrient composition of milk or meat produced by cattle and feed quality will affect the metabolism in the body of livestock that will affect the availability of energy and nutrients for the synthesis of milk or meat components [4].

In this paper, we stress that antioxidant supplementation not only will have a preventive effect on the health of the mother and calf, but in turn can enhance the final product (milk/meat) in line with what is now called *functional foods*, due to the increasing consumer's awareness of the relationship between diet, health, and disease prevention. We will revise how antioxidant supplementation given to cattle for clinical purposes (disease prevention) can also affect the quality of the final product, adding value to production, with benefits not only for the health of animal.

## 2. Antioxidants and Milk Production

The value of a cow lies in its production. And the quality of milk is closely linked to the health of mammary gland. Milk quality is usually defined in terms of mastitis [5]: milk with a low somatic cell count (SCC) and visibly normal appearance (no clots). But, in accordance with Weiss [6], the definition of high-quality milk must be expanded. Thus, the quality of milk can also be based on the amount of antioxidants that it contains, protecting the characteristics of milk lifetime by reducing oxidation.

In previous studies, milk and several fractions thereof were found to have antioxidant properties. For example, milk, kimmed milk, whey and casein inhibit lipid peroxidation and peroxyl/superoxide radicals generation. Furthermore, casein inhibits peroxide and TBARS (thiobarbituric acid reactive substances) formation, and whey inhibits copper-catalysed peroxides, TBARS formation and O_2_ uptake. Lactoferrin can bind iron and inhibit Fe-induced lipid peroxidation and finally hydrolysates from milk, fermented milk, casein, and whey were found to be antioxidative and some of them have been patented. These examples show that several components are active in preventing lipid peroxidation and maintaining milk quality and also point to their potential usage as ingredients in foods to provide products for enhanced consumer health [7]. Depending on their nature, milk antioxidants are divided into protein and non-protein antioxidants [8]. In the first group, some enzymes (superoxide dismutase, catalase and glutathione peroxidase) are included, as well as some proteins (e.g., casein) and peptides. The second group includes vitamins (C and E) and carotenoids, whose knowledge is broad and diverse.

On average, most fluid milk is judged to have a good flavor up to 14 days of storage, but off-flavor of milk is still an important problem [9]. Oxidized flavor (OF) is described as cardboard-like, metallic, or tallowy and can develop over time because of improper storage and handling of the milk. In certain situations, OF can be detected in milk almost immediately following milking. Several authors described that different factors must be determinant for the oxidative stability of milk [6, 10] varying considerably between individual cows and cannot be explained considering only fatty acid composition, or content of low molecular weight antioxidants, such as uric acid, ascorbic acid, and tocopherol (see Table 1).

Antioxidant supplementation comprises less milk waste in the form of free radicals as well as reducing the number of somatic cells in milk. Supplements fed through the diet (vitamin E, vitamin C, carotene, and trace elements such as selenium, zinc or *β*-flavonoids, vitamin A, and manganese—fundamental chain of enzymatic antioxidants—) have been proven useful to reduce the occurrence of udder infections and improve the quality of their production, in terms of fat, protein, and somatic cell count [5, 11], giving to the product an added value as a source of antioxidants for the human diet, with beneficial effects in the gastrointestinal tract and other tissues [8]. An example is the reported functional role of glutathione peroxidase and other Se compounds in milk, preventing lipid oxidation [12]. Later studies [5, 10] showed that cows supplemented with selenium increased the concentrations of the oligoelement in milk, resulting in an increased intake of selenium by humans consuming dairy products. Curiously, these studies reported that milk Se concentrations were twice as high when Se yeast was fed to the cow compared with selenite or selenate.

The antioxidant activity of dairy products has also been considered in fermented milks [8]. Different studies have established the ability of lactic acid bacteria to release certain compounds with antioxidant activity during fermentation of the milk. However, none of these compounds has been isolated and characterized. The characterization of these compounds (Table 2) would be useful as a precursor to the development of new natural dietary antioxidants and the development of new functional foods.

However, one should be cautious about antioxidant supplementation, because as stated by Weiss [6], excessive supplementation may increase oxidative stress, decrease immune function, and increase health problems. Thus, the likelihood that a cow will respond to mineral or vitamin supplementation is a function of the amount of nutrient the cow would consume with an unsupplemented diet.

### 2.1. The Antioxidant Status of the Cow Can Influences the Development of Oxidized Flavor in the Milk

The fatty acid profile of milk fat is a major factor in the development of OF [6] and some antioxidants (e.g., Cu) can increase susceptibility to it [13], especially if the milk is also high in polyunsaturated fatty acids (PUFA's) such as linoleic or linolenic acid [14]. The concentrations of those two PUFA's in milk can be increased by feeding certain oilseeds or rumen protected oils [15, 16]. As the use of these types of products increases as diet supplement, OF may become a larger problem if it is combined with mineral supplementation. This result is in contrast to studies that recommend giving conjugated unsaturated fatty acids, especially linoleic acid (CLA) as a mechanism to increase milk quality [5].

An alternative could be supplementation with the disaccharide trehalose. A recent study [17] stated that trehalose supplementation in the diets of dairy cows produced milk with low lipid peroxide and high antioxidant content, suggesting that trehalose could inhibit OS, enabling the production of low-lipid peroxide and high-antioxidant milk for human consumption and decreasing the unwanted effects of lipid oxidative odor in cow's milk.

Other reviews [18] point out that vitamin E supplementation affects milk quality in two forms: reducing the levels of SCC and the activity of the proteolytic enzyme plasmin in milk (known as *indirect* effect, due to that there is no direct mechanism through which vitamin E causes these effects) and inducing oxidative stability of the milk (known as *direct* effect due to the proportional increment in milk *α*-tocopherol associated to supplementation). These comments seem to disagree with the observations made by Weiss [6] who indicates that usually less than 2% of the vitamin E consumed by a cow is secreted in her milk and the efficiency of transfer decreases as vitamin E intake increases, suggesting that substantial amounts of vitamin E must be consumed by cows to reduce oxidant flavor of milk. It is clear that current available data are not conclusive regarding the amount of dietary vitamin E needed to prevent and maintain milk quality in terms of SCC or oxidation.

Finally, despite the numerous studies describing the beneficial effects of various antioxidants on the quality of milk and its derivatives, still others report that dietary antioxidants have no effect on milk protein, fat, lactose, total solids, and nonfat solids of cows [19] or goats [4] milk.

### 2.2. Production System Also Affects Milk Quality

An increasing number of dairy farms in Europe, New Zealand/Australia, and North America are adapting “lower-input” production methods (also called sustainable farms) similar to those used in organic farming but do not comply with all input restrictions prescribed by organic farming standards.

It has been shown that this system indirectly affects milk quality through the increased amount of antioxidants in milk associated with pasture consumption [20] and is strongly linked to the stage and length of the grazing period and diet composition, which will influence subsequent processing, sensory, and potential nutritional qualities of the milk. 

For these reasons, there is growing interest in the biodiversity of pastures, because they included some microconstituents (phenolic compounds, terpenes, and carotenoids) that contribute to the taste and nutritional properties of milk and cheese [21] in combination with their antioxidative properties (specially for polyphenols and within them, dihydroxycinnamic derivatives). Compared with inorganic supplements, plant-derived products have proven to be natural, less toxic, and residue free and are thought to be ideal growth promoters for both milk and beef productions [22].

Several studies have highlighted the richness in soluble phenolics of the main dicotyledon plants, such as *Tragopogon pratensis*, *Knautia arvensis,* or *Alchemilla xanthochlora*, and isoflavonoids and hydroxycinnamic acids were characterised in some forage plants. On the other hand, permanent pasture is characterized by its botanical biodiversity and, consequently, with high polyphenol content, and that warm temperatures (spring-summer) have a positive effect on the polyphenolic content [21].

## 3. Antioxidants and Meat Quality

**Figure 1:**
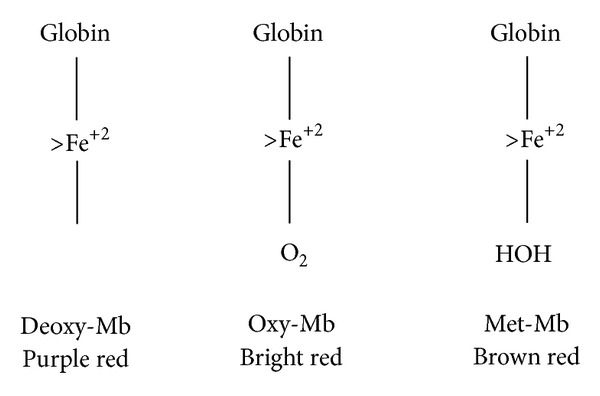
Colour of meat and associated forms of mioglobin [23]. During handling, processing and storage of fresh meat, released endogenous iron is partially responsible for the catalysis of lipid oxidation [25].

Meat can be defined as the product that results from the continuous changes that occur in muscle after the death of the animal. The principal factors determining the organoleptic quality of meat are tenderness, colour, and flavour, the latter being composed in turn of the two distinct factors taste and odor [23]. The color of meat depends on different factors such as haeminic pigments (myoglobin), their pH, and the chemical state of the pigments. Reduced (or deoxy) myoglobin is the purple pigment of deep muscle (Figure 1). Sensory analysis showed that high-grain diets produced more acceptable or more intense flavor in red meats than low-energy forage or grass diets [24]. Nevertheless, as we see, their functional properties as nutrient are quite different.

Oxidation manifests as a conversion of the red muscle pigment myoglobin to brown metmyoglobin and the development of rancid odors and flavors from the degradation of the polyunsaturated fatty acids in the tissue membranes [26, 27]. In meat, antioxidant defenses are composed of non-enzymatic water and lipid soluble compounds like vitamins E and C, carotenoids, ubiquinols, polyphenols, cellular thiols, and enzymes like superoxide dismutase, catalase, and glutathione peroxidase [24].

Lipid oxidation in meat increases after 4 or 7 days of storage; although synthetic antioxidants are widely used in meat industry, the consumer concern over their toxicity initiated the search for natural sources of antioxidants [28]. It is well known that oxidative stability in meat can be enhanced through refrigerated meat storage either for fresh or aged beef and is associated with the deterioration of red color and the formation of metmyoglobin [25]. Some studies have demonstrated that meat shelf-life and quality can be improved by natural antioxidants added in the preslaughter stages, incorporating natural antioxidants in animal diets. Thus, among the positive effects of natural antioxidants on meat characteristics are retarding lipid oxidation, color loss, and microbial growth [25, 27].

### 3.1. Lipid Stability Determines Meat Oxidation

**Figure 2:**
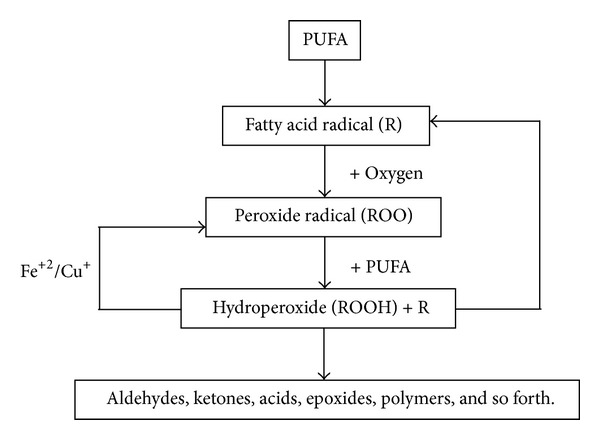
Lipid oxidation is an autocatalytic process that occurs in food and biological membranes [23]. Aldehydes produced are the major contributors to off-flavors of beef [24].

Lipid oxidation is the limiting factor for PUFAs to serve as nutritionally beneficial lipids in functional foods (see Figure 2). In cattle, their concentrations in total lipids are low because of hydrogenation in the rumen which converts a high proportion of polyunsaturated fatty acids from forage or concentrate diets into saturated fatty acids or unsaturated fatty acids with fewer double bonds [26]. The main conjugated linoleic acid (CLA) isomer in beef is CLA *cis-9, trans-11* and it is mainly associated with the triacylglycerol lipid fraction and therefore is positively correlated with level of fatness. The level of this PUFA is related to (1) the amount of this isomer produced in rumen and (2) the synthesis in the tissue by delta-9-desaturase from ruminally produced *trans*vaccenic acid [29].

In addition, the fatty acid compositions of concentrate (grain-based) and forage (grass-based) diets are quite different and lead to different fatty acid compositions in tissues. Different studies [24–26] have demonstrated that increasing the nutritional *α*-linolenic acid resulted in increased content of this PUFA and its longer chain derivate eicosapentaenoic acid in beef muscle and adipose tissue. Grass or concentrates containing linseed are sources of nutritional *α*-linolenic acid; in addition, grass feeding also increases docasahexaenoic acid. These findings are explained by the fact that *α*-linolenic acid is the major fatty acid in grass lipids whereas cereals and the oil seeds used in concentrate diets are major sources of linoleic acid. These differences between grain and forage-based diets are largely responsible for the differences in volatile composition and hence the flavor of beef finished on these diets. Therefore from a nutritional standpoint, grass-fed beef will provide approximately 123 mg CLA from a standard meat product at 10% fat whereas the same product from grain-fed beef would provide 48.3 mg [24].

Nevertheless, meat oxidation is not only due to the lipids instability; the presence of transition metals such as Fe and Cu (given in many cases as mineral supplementation) favors the formation of highly reactive free radicals in meat [23]. Other factors that accelerate meat oxidation are the conditions of slaughter (stress, pH, temperature of the carcass, and electrical stimulation) or rupture of the integrity of muscle membranes (mechanical deboning, grinding, processing, and cooking) [30].

### 3.2. Vitamin Supplementation as a Tool for Preventing Meat Oxidation

Dietary antioxidants can be delivered to the muscle where, together with the native defense systems, they counteract the action of prooxidants [24]. Synthetic antioxidants have been used to delay or minimize oxidative deterioration of foods, such as butylated hydroxyanisole (BHA), butylated hydroxytoluene (BHT), and tertiary butyl hydroquinone (TBHQ), but consumers rejected synthetic antioxidants because of their carcinogenicity [27]. Therefore, recent research has been directed towards the supplementation of vitamins with antioxidant effect either directly in the diet offered to the animals or by adding it to the meat after slaughter.

Vitamin E (*α*-tocopherol) is the most frequently used lipid soluble free radical scavenger administered as nutritional supplement [5, 28], contributing to the stability of beef muscle [23]. Early-studies [31] reported that the post-mortem addition of vitamin E in beef was less effective in delaying lipid oxidation than dietary supplementation. *α*-Tocopherol is the compound usually identified as vitamin E, although other tocopherols also present some vitamin E activity: D-*α*-tocopherol, D-*β*-tocopherol, D-*γ*-tocopherol, D-*δ*-tocopherol, and D-*α*-tocotrienol showing, respectively, 1.49, 0.75, 0.30, 0.15, and 0.45 units of activity [24]. Meat derived from pasture feeding is associated with more antioxidants in the form of D-*α*-tocopherol, carotenoids, and flavonoids [26], which stabilize the fatty acids [32]. Dietary vitamin E supplementation results in elevated concentrations of *α*-tocopherol within cell membranes, increasing the days of retail display life without compromising microbiological quality by preventing the oxidation of membrane phospholipids during storage which inhibits the passage of sarcoplasmic fluid through the muscle cell membrane [26, 28, 33, 34]. First studies [35] have shown that daily supplementation of concentrate diets with 2500 mg vitamin E for 40 d resulted in a 7–10 d extension of colour shelf-life when beef steaks were displayed in modified-atmosphere packs. A trial published in 2010 showed that supranutritional supplementation of grain fed cattle with vitamin E did not affect meat redness or stability compared with that from nonsupplemented cattle, when viewed over a 7 d period of aerobic storage. The stability and improvement in meat color by vitamin E were principally due to its ability to prevent the oxidation of myoglobin and/or oxymyoglobin to metmyoglobin [28].

Nevertheless, concentrations of endogenous antioxidants depend not only on diet, but also on animal species and muscle type [26, 30, 36], and other authors [24] have also stated that the magnitude of benefits resulting from vitamin E supplementation in finishing diets may widely vary, due to the basal diet (quality of pasture, natural grazing, grass silage, and supplement nature) offered to cattle.

When vitamin E is combined with Se supplementation, researchers have observed that in muscle, the antioxidant functions of vitamin E and Se persist after slaughter and delay the onset of oxidation reactions in meat and meat products, demonstrating also that muscle Se levels respond to dietary Se supplementation in beef cattle [5].

In addition to *α*-tocopherol, pasture supplied *β*-carotene (pro-vitamin A) is incorporated to different muscles, such as *M. longissimus dorsi*, *M. semimembranosus, M. gluteus medius,* and *M. psoas major *at levels significantly higher than those for beef cattle [25], although its antioxidant properties appear to be lower than those recorded for vitamin E; in fact, several reports presented that auto-oxidation and the oxidative metabolites of *β*-carotene can act as propagators of free-radical formation. Carotenoids cooperate with tocopherols in the radical scavenging capacity within the inner part of lipid membranes, preventing tissue damage, although the antioxidant capacity of *β*-carotene combined with *α*-tocopherol seems to be inferior to *α*-tocopherol alone because its auto-oxidation weakened the effect of *α*-tocopherol [34].

However, research was performed with other vitamins or their precursors: a vitamin C solution of sodium ascorbate injected in beef was also effective in improving color stability and extending the meat's retail display life [37]. Vitamin C is involved mainly during regeneration (reduction) of tocopheroxyl (oxidized form of vitamin E) obtained through the antiradical activity of tocopherol, suggesting that these two molecules could act synergistically [24]. Its presence in the cytoplasm side of cell membranes, close to tocopherol molecules, could help to maintain the antioxidant status within the tissue. It has been reported [25] that pasture feeding could enhance ascorbic acid concentration in muscle tissue, whereas others have shown that postmortem addition of vitamin C to ground beef was effective in delaying red color deterioration in grain or grass produced meat.

### 3.3. Plant Extracts as a Natural Way to Prevent Meat Oxidation

In recent years, substances derived from the plants have been successfully used to reduce lipid oxidation in meat products [38]; their antioxidant properties are apparently related to their phenolic content [28]. The potential antioxidant role of natural plant extracts has been considered in different studies, suggesting an interesting field to explore, in line with the consumer's criteria related to any additive: safety [22] (see Table 3).

Many herbs, spices, and their extracts have been added to a variety of foods to improve their sensory characteristics and extend shelf-life. Herbs of the *Lamiaceae* family, mainly oregano (*Origanum vulgare *L.), rosemary (*Rosmarinus officinalis *L.), and sage (*Salvia officinalis *L.), have been reported as having significant antioxidant capacity [27] attributable to three mechanisms: free-radical scavenging activity, transition-metal-chelating activity, and/or singlet-oxygen-quenching capacity. On the other hand, it has been described that the properties of curcumin are a good antioxidant that inhibits lipid peroxidation in rat liver microsomes, erythrocyte membranes, and brain homogenates, suggesting also that the curcuma antioxidants are more potent compared to vitamin E [28].

It has been observed that surface application of vitamin C, taurine, rosemary, vitamin E, and combinations of the last three with vitamin C has a positive effect on oxidative stability of beef steaks packaged in modified atmosphere [39].

Meat quality can be improved by incorporating these natural antioxidants into animal diets, adding these compounds onto the meat surface, or using active packaging. Some authors have reported that natural antioxidants have no effect on sensory characteristics of meat. There are studies that demonstrate that the addition of essential oil compounds to the diet of growing lambs (carvacrol and cinnamaldehyde) did not affect the sensory characteristics of sirloins [40]. The only evidence of the effect of natural antioxidants on inhibiting off-odor formation and discoloration of meat is active packaging [41].

Reduction of meat oxidation during refrigeration was obtained adding oregano and sage essential oils to beef meat [42] or even spraying a rosemary and vitamin C solution onto the surface [43]. In addition, dietary incorporation of oregano, rosemary, and sage essential oils can delay lipid oxidation in meat during refrigerated and frozen storage [27].

Lower oxidant formation in dietary oregano essential oil treatments is probably the result of the presence of oregano antioxidant compounds, which might be absorbed into the circulatory system after ingestion, distributed, and retained in muscle and other tissues [44].

## 4. Conclusion

In the modern bovine farming systems, where the main objective is to obtain products of high quality (milk or meat); the concept of quality do not only include a safe product for the consumer, but also the use of farming practices that respect animals' health, either in intensive or extensive systems. Antioxidant supplementation would enhance the health of the cow in a sensitive stage such as the transition period but also can have an additional value, giving to the final product (milk/meat) value added that benefits consumer's health.

It seems that, considering the reviewed studies, animals kept in sustainable conditions, where their production is in line with the physiological processess associated with lactation and/or growth, provide the most complete human foods from the standpoint antioxidant.

Grazing animals and their diet rich in plant extracts are reflected in the production of milk and meat that responds perfectly to the concept of functional food in the human diet.

## Figures and Tables

**Table 1:** Antioxidant systems found in mammalian cells [6].

Component (location in cell)	Nutrients involved	Function
Superoxide dismutase (cytosol)	Cu and Zn	An enzyme that converts superoxide to hydrogen peroxide
Superoxide dismutase (mitochondria)	Mn and Zn	An enzyme that converts superoxide to hydrogen peroxide
Ceruloplasmin	Cu	An antioxidant protein may prevent copper from participating in oxidation reactions
Glutathione peroxidase (cytosol)	Se	An enzyme that converts hydrogen peroxide to water
Catalase (cytosol)	Fe	An enzyme (primarily in liver) that converts hydrogen peroxide to water
*α*-Tocopherol (membranes)	Vitamin E	Breaks fatty acid peroxidation chain reactions
*β*-Carotene (membranes)	Vitamin A	Prevents initiation of fatty acid peroxidation chain reactions

**Table 2:** Fermented milks with antioxidant activity [8].

Type of milk	Bacterial strain	Type of assay	Antioxidant mechanism
Bovine milk	*Lactobacillus delbrueckii sp. bulgaricus *	*In vitro *	DPPH radical chelating activity
Bovine milk	*Lactobacillus rhamnosus *	*In vitro *	Chelating activity of superoxide anion; inhibition of lipid peroxidation
Buffalo whey	*Streptococcus thermophilus* or *Lactobacillus delbrueckii sp. bulgaricus *	*In vitro *	Inhibiting the decomposition of peroxides; chelation of transition metals
Bovine whey	n.r.	*In vitro *	Reduction of oxidative stress in rats with dietary vitamin E deficiency
Goat milk	*Lactobacillus fermentum *	*In vivo *(humans)	Antiatherogenic effects in healthy humans

DPPH: free radical diphenyl picrylhydrazyl; n.r. not reported.

**Table:** (a)

Common name	Scientific name	Main compounds (class)
Alfalfa	*Medicago sativa *	Coumestrol (flavonoid)
Allspice	*Pimenta dioica *	Eugenol (EO)
Apple	*Malus sylvestris *	Phloretin (flavonoid)
Bael tree	*Aegle marmelos *	Limonene (terpene)
Barberry	*Berberis vulgaris *	Berberine (alkaloid)
Basil	*Ocimum basilicum *	Linalool (terpene); estragol (EO)
Bay	*Laurus nobilis *	Linalool (terpene); cineole (EO)
Betel pepper	*Piper betel *	Eugenol (EO)
Black pepper	*Piper nigrum *	Piperine (alkaloid)
Brazilian pepper tree	*Schinus terebinthifolius *	Terebinthina (terpene)
Burdock	*Arctium lappa *	Cinarin, Quercetin; caffeic acid (phenols)
Caraway	*Carum carvi *	Carvone; limonene; germacrene (terpenes)
Cascara sagrada	*Rhamnus purshiana *	Anthraquinone (phenolic: quinone)
Ceylon cinnamon	*Cinnamomum verum *	Pinene (terpene); cinnamaldehyde; eugenol (EOs)
Chamomile	*Matricaria chamomilla *	Anthemic acid (phenolic)
Chili peppers, paprika	*Capsicum annuum *	Capsaicin (terpene)
Clove	*Syzygium aromaticum *	Eugenol (EO)
Dill	*Anethum graveolens *	Carvone; limonene (terpene)
Echinacea	*Echinacea angustifolia *	Polyenes (polyacetylenes); Cyarin (phenolic); tussilaginea (alkaloid)
Garlic	*Allium sativum *	Allicin; Ajoene (S-terpene)
Ginseng	*Panax notoginseng *	Ginsenoside (saponin)
Glory lily	*Gloriosa superba *	Colchicine (alkaloid)
*Goldenseal *	*Hydrastis canadensis *	Berberine (alkaloid)
Gotu kola	*Centella asiatica *	Asiaticoside (terpene)
Grapefruit peel	*Citrus paradise *	Ocimene *β* (terpene)
Green tea	*Camellia sinensis *	Catechin (flavonoid)
Hops	*Humulus lupulus *	Lupulone (phenolic), humulone (terpene)
Horseradish	*Armoracia rusticana *	Kaempferol (flavonol)

EO: Essential oil.

**Table:** (b)

Common name	Scientific name	Main compounds (class)
Legume	*Millettia thonningii *	Alpinumisoflavone
Lemon balm	*Melissa officinalis *	Tannins; carveol (EO); saponins; citronellol (terpene)
Lemongrass	*Cymbopogon citratus *	Citral (EO)
Lemon verbena	*Aloysia triphylla *	
Mace, nutmeg	*Myristica fragrans *	Sabinene (terpene)
Oak	*Quercus rubra *	Quercetin (flavonoid)
Olive oil	*Olea europaea *	Oleuropein (phenolics)
Onion	*Allium cepa *	
Orange peel	*Citrus sinensis *	Limonene (terpene)
Oregon grape	*Mahonia aquifolium *	Berberine (alkaloid)
Papaya	*Carica papaya *	Papain (polypeptide)
Peppermint	*Mentha piperita *	Menthol (EO)
Purple prairie clover	*Petalostemum *	Petalostemumol (flavonol)
Quinina	*Cinchona sp. *	Quinine (alkaloid)
Rauvolfia, Chandra	*Rauvolfia serpentine *	Reserpine (alkaloid)
Rosemary	*Rosmarinus officinalis *	Rosmarinic acid (phenolic); carnosol (terpene)
Sainfoin	*Onobrychis viciifolia *	Tannins
Savory	*Satureja montana *	Carvacrol (terpenoid)
Senna	*Cassia angustifolia *	Rhein (phenolic quinine)
Tansy	*Tanacetum vulgare *	Chrysanthenyl acetate (EO)
Tarragon	*Artemisia dracunculus *	Caffeic acid (phenolic)
Thyme	*Thymus vulgaris *	Thymol (EO)
Turmeric	*Curcuma longa *	Curcumin (terpene)
Valerian	*Valeriana officinalis *	Linarin (flavone); elemol (terpene)
Willow	*Salix alba *	Salicin (phenolic)
Wintergreen	*Gaultheria procumbens *	Anthocyanins (phenolic)
